# Collision Avoidance Strategy for the Autocrane

**DOI:** 10.3390/s21206746

**Published:** 2021-10-11

**Authors:** Dong Wang, Baochang Liu, Jian Shen, Li Chen, Lydia Zhu

**Affiliations:** 1 School of Instrument Science and Engineering, Southeast University, Nanjing 210096, China; 22017210@seu.edu.cn; 2 NARI Group Corporation, State Grid Electric Power Research Institute, Nanjing 211106, China; shenjian@sgepri.sgc.com.cn (J.S.); chenli1@sgepri.sgc.com.cn (L.C.); 3 NARI Technology Co., Ltd., Nanjing 211106, China; 4 State Key Laboratory of Smart Grid Protection and Control, Nanjing 211106, China; 5 Department of Mechanical Aerospace & Engineering, North Carolina State University, Raleigh, NC 27695, USA; Lzhu6@ncsu.edu

**Keywords:** collision avoidance strategy, boom control, collision urgency evaluate

## Abstract

The collision between the boom of the autocrane and the obstacle may cause serious equipment damages or casualties. With the development of 6G technology, data between multiple autocranes could be shared in real time, which makes it possible to finely control the autocranes. In order to avoid collision accidents, a collision avoidance strategy is proposed in this paper. This strategy focuses on the evaluation of the collision urgency and different evaluation methods are designed to match the three basic exercise modes of the boom. Based on the collision urgency, the control strategy is then put forward to ensure the boom’s safety in every collision risk level. Additionally, two special cases are also considered to guarantee that all parts of the boom, except for the end, will not hit the obstacle. Lastly, a semi-physical testing platform is established to test the performance of the proposed collision avoidance strategy.

## 1. Introduction

The autocrane, which is equipped with the lifting mechanism boom, is widely used for moving heavy objects in the engineering construction industry and in the working condition [1,2]. The crane operator uses a series of transmission devices to drive the boom to move in the space, thereby manipulating the hook to lift heavy objects [3,4]. However, due to the complex operating environment and the negligence of the operator, collision between the boom and the obstacle sometimes occurs. Especially when the steel boom touches the high voltage cable, the collision may result in serious personal injuries and deaths, which should be avoided as much as possible [5,6].

Researchers worldwide have developed many effective methods to make the movement of the boom smoother with easier operation and to reduce the collision risk caused by mis-operation [7,8,9]. Hirokazu Araya [10,11] invented a luffing follow-up control system, which could realize the horizontal movement of the hoisting load by simultaneously controlling the hoisting hoist and luffing hoist on the crawler crane. At the same time, a linear excavation control system for the hydraulic excavator was also proposed and the straight-line excavation of the bucket could be realized by controlling the boom, stick, and bucket cylinder. The Margelus company applied for a patent for the path control and vibration suppression of an articulated ladder or lifting platform. In this patent, a variety of sensors are used to collect signals and input them into the controller for calculation to improve the accuracy of the movement of a single mechanism and suppress the vibration problems during exercise. Additionally, Eckhard [12] used a non-linear control strategy to solve the radial yaw phenomenon caused by the crane in trajectory tracking. Sebastian [13] used the feedforward method to suppress the vibration caused by the single-stroke luffing hydraulic cylinder in the horizontal crane load. Ehsan Maleki [14] used input signal-tuning to suppress the swing caused by the horizontal load of the crane. While the above methods have improved the control accuracy of the boom of the autocrane, the distance between the obstacle and the boom was not considered in any of the control strategies, which should raise the collision risk rapidly when the boom is near the obstacle [15,16].

Thus, in this paper, a two-stage collision avoidance strategy is proposed, which could limit the exercise of the boom, based on both the collision urgency and the distance to the obstacle. In this way, when the boom approaches obstacles, it will go through two processes: speed limit and being forced to stop, which make the movement of the boom smoother while ensuring safety. To identify the actual effect of the strategy, three basic movement modes of the boom are tested in the semi-physical experiments.

The remainder of this paper is organized as follows. The kinematic model of the autocrane is given in Section 2. Section 3 elaborates on the distance-based collision avoidance strategy and in this section, the strategy is divided to fit the three single movements of the boom, which are pitching, stretching, and horizontal rotation. In Section 4, a semi-physical simulation platform is built to verify the correction of the proposed collision avoidance strategy. Furthermore, the simulations and the experiments show the actual effect of the strategy. Section 5 concludes the paper.

## 2. Kinematic Model of the Autocrane

The concise kinematic model is essential in the formulation of the collision avoidance strategy, which is used to describe the relationship between the position of the boom and its control inputs.

### 2.1. Space Position of the Boom

**Figure 1:**
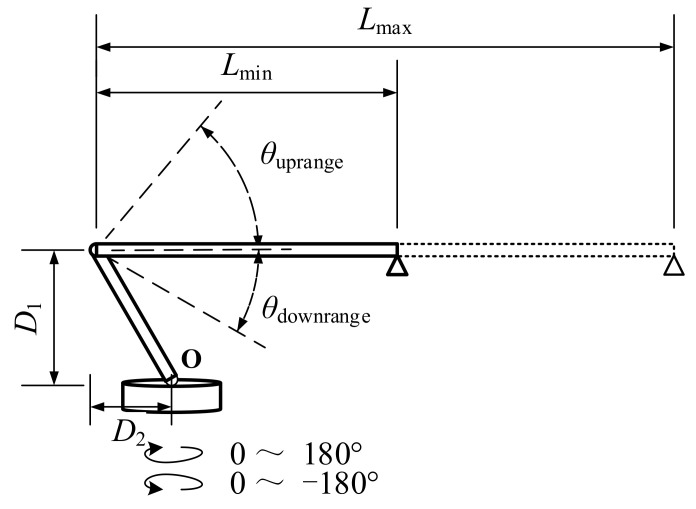
Structure of the autocrane.

The structure of the autocrane could be simplified as shown in Figure 1, in which the vehicle part is ignored [17]. The whole structure could rotate around point *O* and the lengths of $D_{1}$ and $D_{2}$ do not change in the movement of the boom. The boom of the autocrane could stretch between $L_{max}$ and $L_{min}$, and pitch in the range of $\theta_{uprange}+\theta_{downrange}$. The tail end of the boom was marked with a triangle symbol. It is obvious that there are three single modes of motion for the boom, which are pitching, stretching, and horizontal rotation [18].

**Figure 2:**
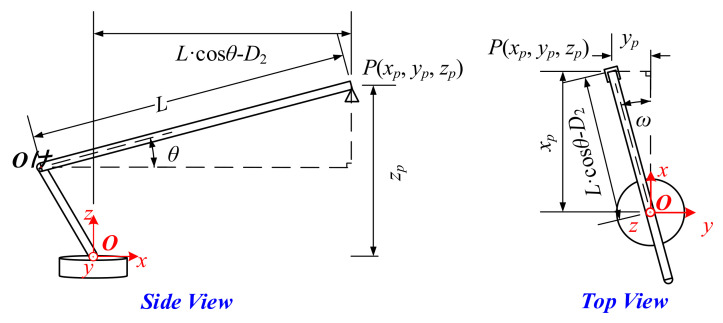
Relationship between the movement of the boom and its spatial position.

In order to describe the relationship between the movements of the boom and its spatial position, which is a coordinate (Oxyz), is formulated in Figure 2. O is fixed at the center of the rotation. The axis Oz is positioned vertically upwards and coincides with the rotation axis. The axis Ox is positioned towards the head of the autocrane and the axis Oy is positioned to the side to constitute a right-handed coordinate. In this way, the position of the boom could be completely determined by the coordinates of points $O^{\prime }$ and P, which are the two end points of the boom, as shown in Equations (1) and (2).

(1) $$O^{\prime }\left(x_{O}^{\prime },y_{O}^{\prime },z_{O}^{\prime }\right)\;:\;\left\{\begin{array}{c} x_{O}^{\prime }=-D_{2}*\cos\omega \\ y_{O}^{\prime }=-D_{2}*\sin\omega \\ z_{O}^{\prime }=D_{1} \end{array}\right.$$

(2) $$P\left(x_{p},y_{p},z_{p}\right)\;:\;\left\{\begin{array}{c} x_{p}=\left(L*\cos\theta -D_{2}\right)*\cos\omega \\ y_{p}=\left(L*\cos\theta -D_{2}\right)*\sin\omega \\ z_{p}=L*\sin\theta +D_{1} \end{array}\right.$$

where $L\;\left(L_{min}\leq L\leq L_{max}\right)$ is the length of the boom. $\theta \;\left(-\theta_{downrange}\leq \theta \leq \theta_{uprange}\right)$ is the pitch angle of the boom and is defined as positive when positioned upwards. $\omega \;\left(-180{}^{\circ}\leq \omega \leq 180{}^{\circ}\right)$ is the horizontal rotational angle of the boom and the positive direction is defined in the counterclockwise direction. The above three parameters are the control amount inputs of the boom and could make the boom stretch, pitch, and horizontally rotate, respectively.

### 2.2. Space Position of the Obstacle

**Figure 3:**
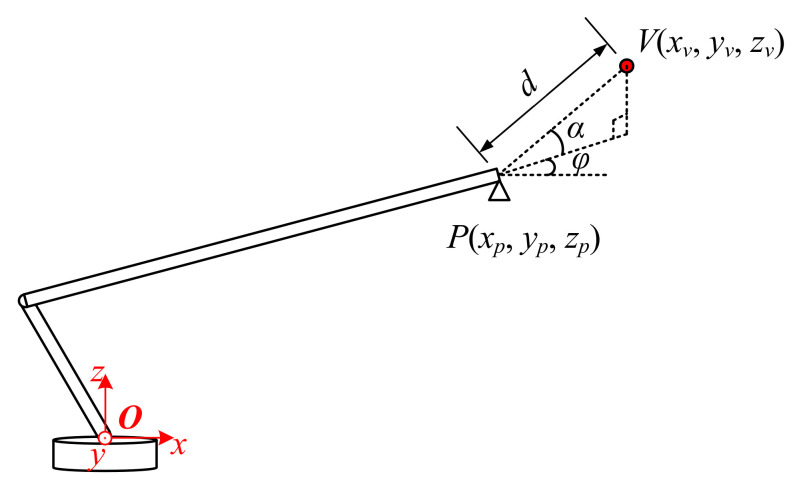
Relative position between the boom and obstacle.

The detection of the obstacle was the premise of collision avoidance. For instance, take the high voltage cable as an example: the available sensors include one or more cameras, an electric field sensor, a millimeter wave radar, and LIDAR [19,20]. These sensors are always fixed on the tail end of the boom for convenience and could obtain the relative position between the boom and obstacle [21], as shown in Figure 3.

Where *d* is the length of line segment *PV*, α and φ are the pitch angle and heading angle of line segment *PV* in the coordinate Oxyz. The above parameters (*d*, α, and φ) could fully describe the relative position between the boom and the obstacle. In this case, the space position of the obstacle $V\left(a^{\prime },b^{\prime },c^{\prime }\right)$ in the coordinate Oxyz is obvious, as shown in Equation (3).

(3) $$V\left(x_{v},y_{v},z_{v}\right)\;:\;\left\{\begin{array}{c} x_{v}=x_{P}+d*\cos\alpha *\cos\varphi \\ y_{v}=y_{P}+d*\cos\alpha *\sin\varphi \\ z_{v}=z_{P}+d*\sin\alpha \end{array}\right.$$

## 3. Collision Avoidance Strategy

**Figure 4:**
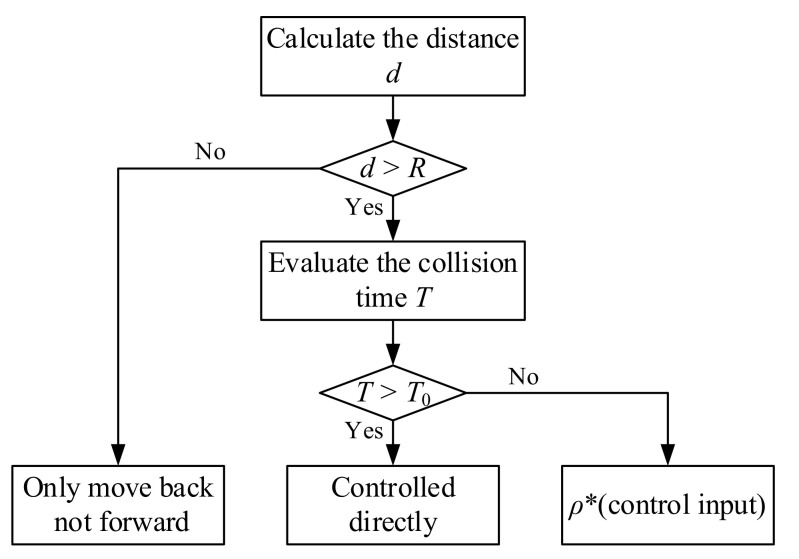
Collision avoidance strategy.

The collision avoidance strategy in this paper is based on both the safety range and the collision urgency. Firstly, a safety radius *R* is defined around the obstacle and the boom is not allowed to enter the range of the radius, which means the boom must stop moving forward and only allow for only moving backwards when the distance to the obstacle (*d* in Figure 3) is less than or equal to the safety radius *R* [22,23]. Secondly, considering that the emergency brake easily causes damage to the boom actuator, a hierarchical control method depending on the collision urgency was designed to make the boom move more smoothly. The collision urgency was evaluated by the time *T* for the boom to reach the safety range, which is defined in Equation (4). It is worth noting that $v_{c}$ is not the real speed of the boom but rather is the speed corresponding to the operator’s input. When *T* is greater than threshold *T*_0_, which means there is no collision in the near future, the boom is directly controlled by the operator of the autocrane. As long as the detecting *T* is less than *T*_0_, meaning the boom has a higher risk of collision, the control value input by the operator will be multiplied by the coefficient ρ (0<ρ<1) before being sent to the boom controller. Figure 4 explains the above strategy. In taking the three movement modes of the boom into account, the specific calculation methods of the parameter *T* are different, which will be illustrated in the rest of this section.

(4) $$T=\frac{d-R}{v_{c}}$$

**Figure 5:**
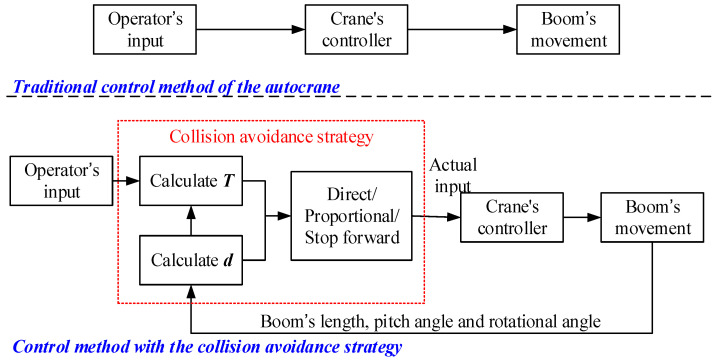
Control method with and without the collision avoidance strategy.

Figure 5 shows how the collision avoidance strategy improves the traditional boom control method. In the traditional strategy, the operator controls the boom directly through the crane’s controller, thus, if the operator does not take braking measures, there is a risk of the boom colliding with obstacles. However, in this paper, the proposed strategy determines whether the operator’s control input is directly proportional or otherwise will stop moving forward, as delivered to the controller according to the calculation of *T* and *d*. This means that the operator can control the boom in any way and the boom will not collide with obstacles.

### 3.1. Collision Urgency Evaluation for Stretching

**Figure 6:**
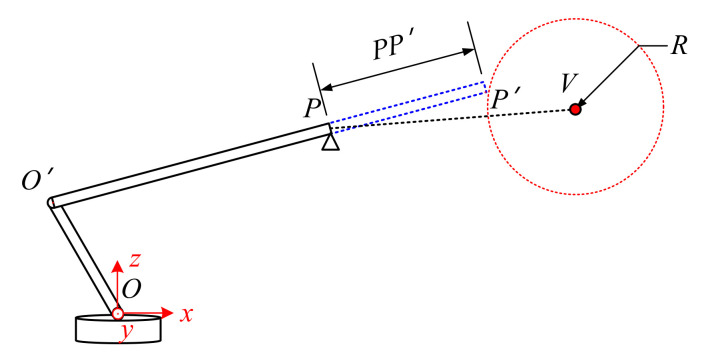
Collision urgency evaluation for stretching.

As shown in Figure 6, the boom could only stretch in this situation. It is obvious that the end of the boom is not allowed to break though the surface of the safety sphere, thus the collision condition can be described as in Equation (5). When the boom is stretching at a constant speed $V_{L}$, the coordinates of point *P′* could be provided, as in Equation (6).

(5) $${\left(x_{v}-x_{p^{\prime }}\right)}^{2}+{\left(y_{v}-y_{p^{\prime }}\right)}^{2}+{\left(z_{v}-z_{p^{\prime }}\right)}^{2}=R^{2}$$

(6) $$\left\{\begin{array}{c} x_{p^{\prime }}=x_{P}+V_{L}T\cos\theta *\cos\omega \\ y_{p^{\prime }}=y_{P}+V_{L}T\cos\theta *\sin\omega \\ z_{p^{\prime }}=z_{P}+V_{L}T\sin\theta \end{array}\right.$$

Substitute Equation (6) into Equation (5): the parameter *T*, which represents the collision urgency, could be obtained by solving a quadratic equation in one variable. Although the equation supports having two solutions for entering and leaving the safety range, *T* is obviously corresponding to the entry point *P′*.

### 3.2. Collision Urgency Evaluation for Pitching

**Figure 7:**
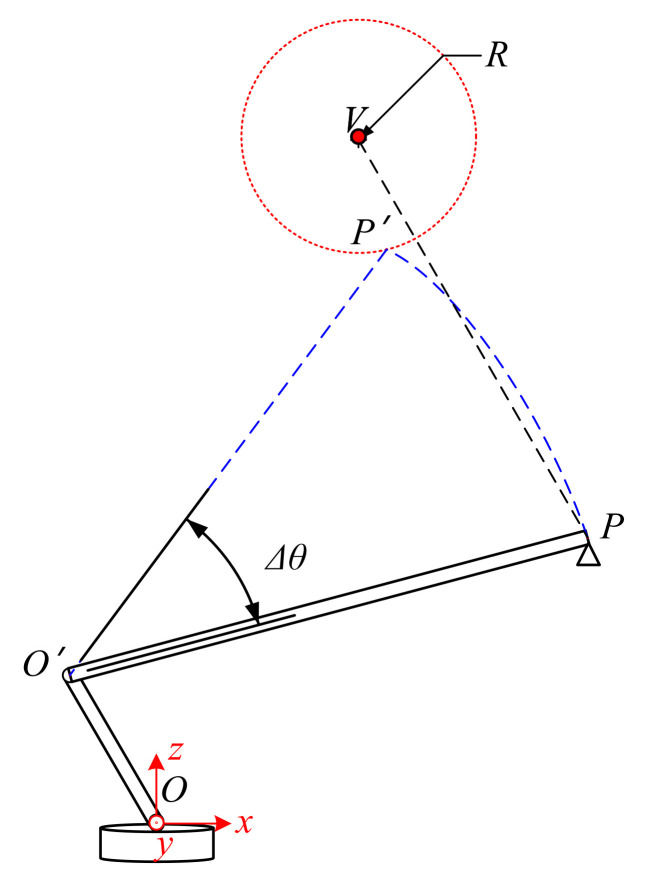
Collision urgency evaluation for pitching.

Figure 7 shows the possible collision situation in the pitching of the boom. As in the stretching, when a collision occurs, the relationship between *P′* and *V* still satisfies Equation (5). In this case, however, the coordinate of *P′* should be described in Equation (7).

(7) $$\left\{\begin{array}{c} x_{p^{\prime }}=\left[L*\cos\left(\theta +V_{\theta }T\right)-D_{2}\right]*\cos\omega \\ y_{p^{\prime }}=\left[L*\cos\left(\theta +V_{\theta }T\right)-D_{2}\right]*\sin\omega \\ z_{p^{\prime }}=L*\sin\left(\theta +V_{\theta }T\right)+D_{1} \end{array}\right.$$

where $V_{\theta }$ is the pitch rate of the boom, which satisfies $\Delta \theta =V_{\theta }T$. Additionally, the estimated collision time *T* could be calculated by solving Equations (5) and (7).

### 3.3. Collision Urgency Evaluation for Horizontal Rotation

**Figure 8:**
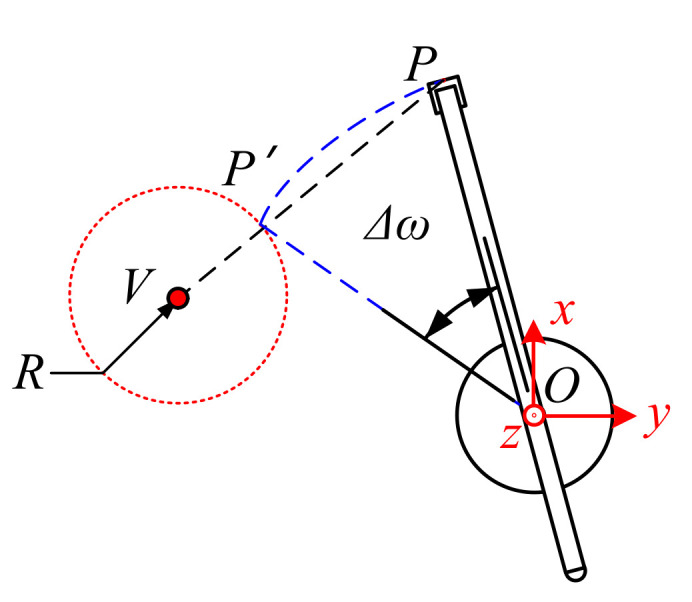
Collision urgency evaluation for horizontal rotation.

The situation of the horizontal rotation of the boom is shown in Figure 8. When the end of the boom touches the surface of the safety sphere, the coordinate of point *P′* is provided, as in Equation (8).

(8) $$\left\{\begin{array}{c} x_{p^{\prime }}=\left(L*\cos\theta -D_{2}\right)*\cos\left(\omega +V_{\omega }T\right)\\ y_{p^{\prime }}=\left(L*\cos\theta -D_{2}\right)*\sin\left(\omega +V_{\omega }T\right)\\ z_{p^{\prime }}=L*\sin\theta +D_{1} \end{array}\right.$$

where $V_{\omega }$ is the rotational speed of the boom, which satisfies $\Delta \omega =V_{\omega }T$. In this way, the time *T* could be obtained from Equations (5) and (8).

### 3.4. Special Cases

**Figure 9:**
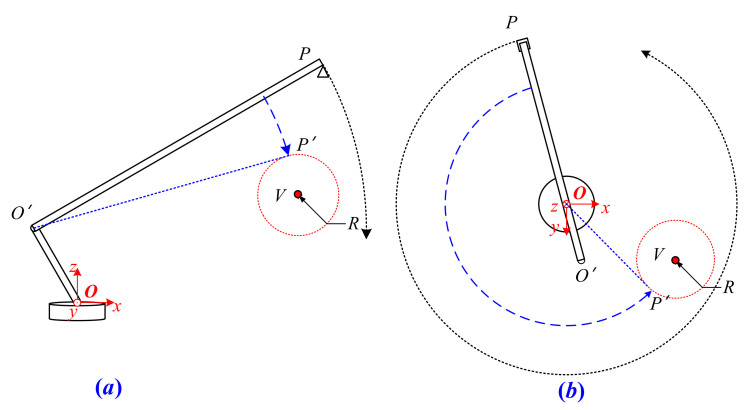
Collision urgency evaluation for special cases: (**a**) special pitching cases, (**b**) special rotating cases.

In addition to the above three cases, it is worth noting that the end of the boom is not the only place where it may collide with obstacles. When the boom is pitching or rotating horizontally, the middle of the boom might be the first to enter the range of the safety sphere, as shown in Figure 9. Under this circumstance, the collision point could be any part of the boom, thus the distance *D’* from point *V* to a straight line *O’P* in space should be checked.

Specifically, for Figure 9a, since the boom engages in a pitching motion, the point *O′* does not move and the line of the boom could be described as in Equation (9).

(9) $$\frac{x-x_{O}^{\prime }}{X_{a}}=\frac{y-y_{O}^{\prime }}{Y_{a}}=\frac{z-z_{O}^{\prime }}{Z_{a}}$$

where ${\left[X_{a},\;Y_{a},\;Z_{a}\right]}^{\prime }$ is the direction vector of the boom, which satisfies Equation (10).

(10) $$\left\{\begin{array}{c} X_{a}=L*\cos\left(\theta +V_{\theta }T\right)*\cos\omega \\ Y_{a}=L*\cos\left(\theta +V_{\theta }T\right)*\sin\omega \\ Z_{a}=L*\sin\left(\theta +V_{\theta }T\right) \end{array}\right.$$

When the boom reaches the safety sphere, *D′* equals to *R*, as in Equation (11).

(11) $$D^{\prime }=\frac{\sqrt{\left|\begin{array}{cc} y_{v}-y_{O}^{\prime } & z_{v}-z_{O}^{\prime }\\ Y_{a} & Z_{a} \end{array}\right|+\left|\begin{array}{cc} z_{v}-z_{O}^{\prime } & x_{v}-x_{O}^{\prime }\\ Z_{a} & X_{a} \end{array}\right|+\left|\begin{array}{cc} x_{v}-x_{O}^{\prime } & y_{v}-y_{O}^{\prime }\\ X_{a} & Y_{a} \end{array}\right|}}{\sqrt{X_{a}{}^{2}+Y_{a}{}^{2}+Z_{a}{}^{2}}}=R$$

The estimated collision time *T* could be calculated by Equations (10) and (11).

Similarly, for Figure 9b, the fixed-point *O_b_* in rotating motion is the intersection point of the boom and axis Oz, and the coordinate of this point is shown in Equation (12). The straight line in the space where the boom is located can be expressed as Equation (13).

(12) $$\left\{\begin{array}{c} x_{Ob}=0\\ y_{Ob}=0\\ z_{Ob}=D_{1}+D_{2}\tan\theta \end{array}\right.$$

(13) $$\frac{x-x_{Ob}}{X_{b}}=\frac{y-y_{Ob}}{Y_{b}}=\frac{z-z_{Ob}}{Z_{b}}$$

where ${\left[X_{b},\;Y_{b},\;Z_{b}\right]}^{\prime }$ is the direction vector of the boom, which satisfies Equation (14).

(14) $$\left\{\begin{array}{c} X_{a}=\left(L*\cos\theta -D_{2}\right)*\cos\left(\omega +V_{\omega }T\right)\\ Y_{a}=\left(L*\cos\theta -D_{2}\right)*\sin\left(\omega +V_{\omega }T\right)\\ Z_{a}=L*\sin\theta -D_{2}\tan\theta \end{array}\right.$$

In this case, when the boom reaches the safety sphere, *D’* equals to *R*, as in Equation (15).

(15) $$D^{\prime }=\frac{\sqrt{\left|\begin{array}{cc} y_{v}-y_{Ob} & z_{v}-z_{Ob}\\ Y_{b} & Z_{b} \end{array}\right|+\left|\begin{array}{cc} z_{v}-z_{Ob} & x_{v}-x_{Ob}\\ Z_{b} & X_{b} \end{array}\right|+\left|\begin{array}{cc} x_{v}-x_{Ob} & y_{v}-y_{Ob}\\ X_{b} & Y_{b} \end{array}\right|}}{\sqrt{X_{b}{}^{2}+Y_{b}{}^{2}+Z_{b}{}^{2}}}=R$$

Additionally, time *T* could be obtained by Equations (14) and (15).

## 4. Semi-Physical Experiments

**Figure 10:**
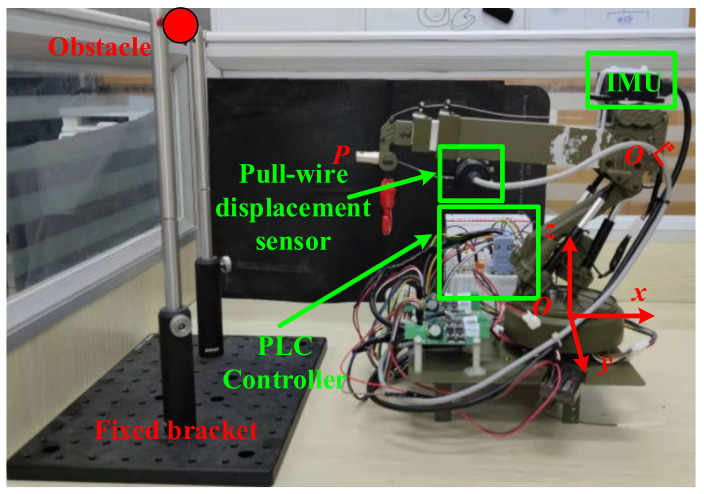
Semi-physical testing platform.

In order to test the performance of the proposed collision avoidance strategy, a semi-physical testing platform was established, as shown in Figure 10. The electric control boom model, which has three movable degrees of freedom (pitching, stretching, and horizontal rotation) was used to simulate the real boom on the autocrane. Its movement was controlled by the PLC controller. The sensors on the model contribute the spatial location of the boom. The pull-wire displacement sensor fixed on the end of the boom could measure the length of the boom (*L*). At the same time, the IMU gave the pitching and rotational angle (θ and ω) of the boom. Additionally, the horizontal position and height of the obstacle could be adjusted by the fixed bracket and support rod.

In this testing platform, some of the structural parameters could be determined before the experiments. Specifically, $D_{1}=130\;\mathrm{mm}$, $D_{2}=67.5\;\mathrm{mm}$, $L_{max}=462\;\mathrm{mm}$, $L_{min}=240\;\mathrm{mm}$, $\theta_{uprange}=50{}^{\circ}$, and $\theta_{downrange}=30{}^{\circ}$.

Furthermore, the parameters for the collision avoidance strategy were also set, such as the safety radius *R* = 50 mm, collision time threshold *T*_0_ = 5 s, and control coefficient ρ = 0.3. Under the above premises, the stretching, pitching, and rotation tests were carried out.

### 4.1. Stretching Test

**Figure 11:**
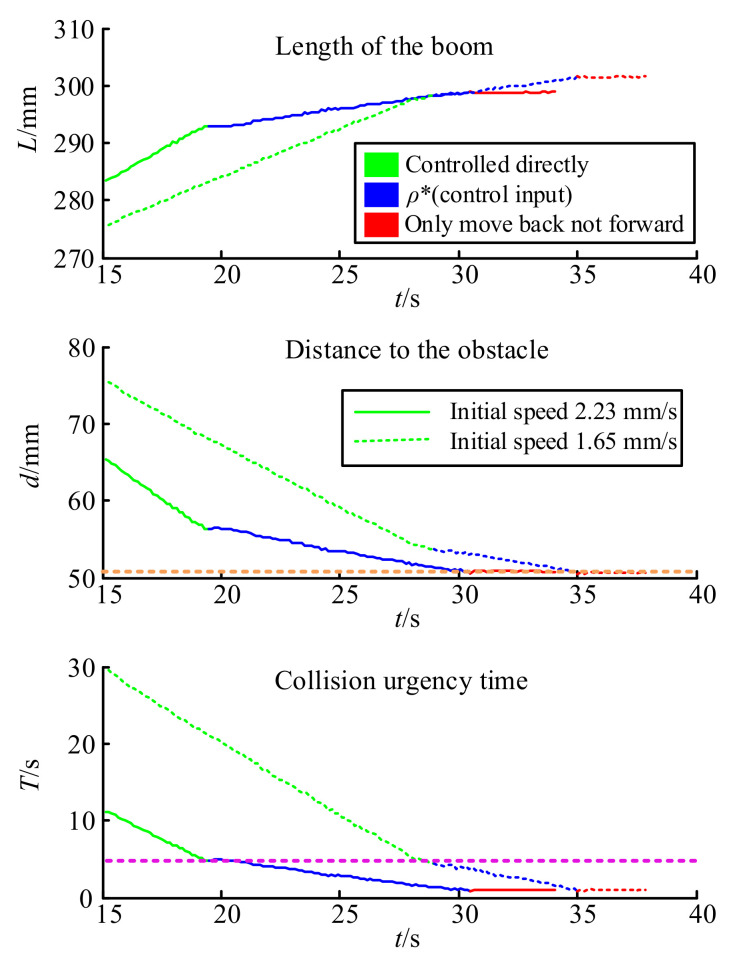
Result of the stretching test.

In the stretching test, the location of the obstacle in coordinate *Oxyz* was set as (273, 0, 130) and *Oxyz* had a unit length of 1 mm. The initial states of the boom were *L* = 240 mm, θ=0°, ω=0°. It is supposed that the operator controls the boom to extend at a constant speed and both the real change of the boom length and the distance to the obstacle are shown in Figure 11.

In Figure 11, the solid and dashed lines represent different original extending speeds, which are 2.23 mm/s for the solid line and 1.65 mm/s for the dashed line. By comparing the time and position of the boom when entering the control intervention phase, it can be found that at different initial speeds, the collision urgency time *T* of the proportional control stage (rose dotted line) and the distance to the obstacle of the forward limit stage (orange dotted line) were always the same, which is consistent with the strategy design.

### 4.2. Pitching and Rotating Test

**Figure 12:**
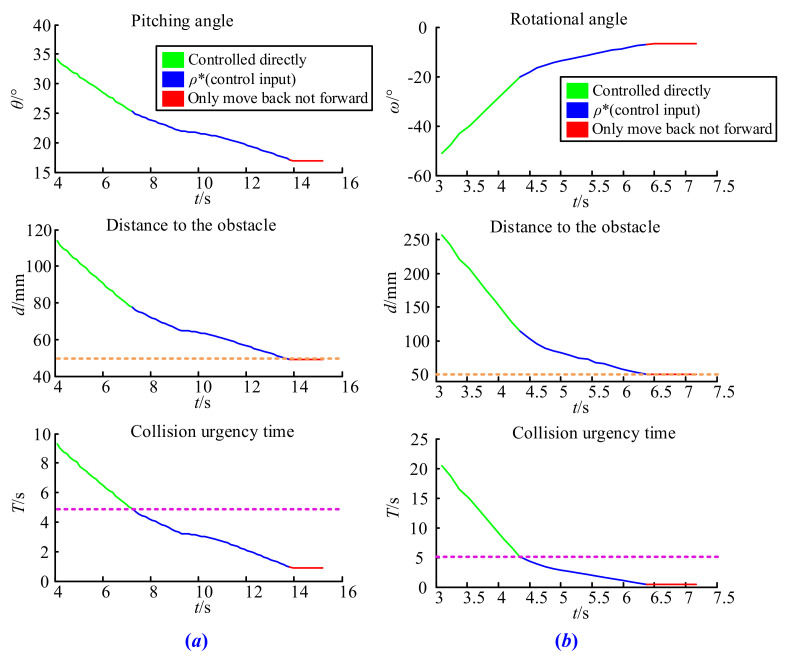
Result of the pitching and rotating test: (**a**) pitching test, (**b**) rotating test.

The initial parameters in the pitching test were set as *V*(180, 0, 180), *L* = 240 mm, θ=60°, and ω=0°, and for the rotating test, *V*(180, 50, 130), *L* = 240 mm, θ=0°, and ω=−63°. The testing results are shown in Figure 12a,b. It is obvious that under the proposed collision avoidance strategy, both the pitching rate and the rotational speed of the boom started to decelerate when the collision urgency time *T* was less than 5 s (rose dotted line) and it was not allowed to move forward when the end of the boom touched the surface of the safety sphere, which means that its distance to the obstacle equaled to 50 mm (orange dotted line).

### 4.3. Special Case Test

When the boom is pitching, if the length of vector *O’V* is less than the length of vector *O’P*, it belongs to the special case discussed earlier. Similarly, when the projection length of vector *OV* in the horizontal plane is less than that of vector *OP*, the end of the boom does not easily collide with obstacles, either. Under these special cases, the time *T* represents the shortest time for collision with obstacles at any position of the boom.

**Figure 13:**
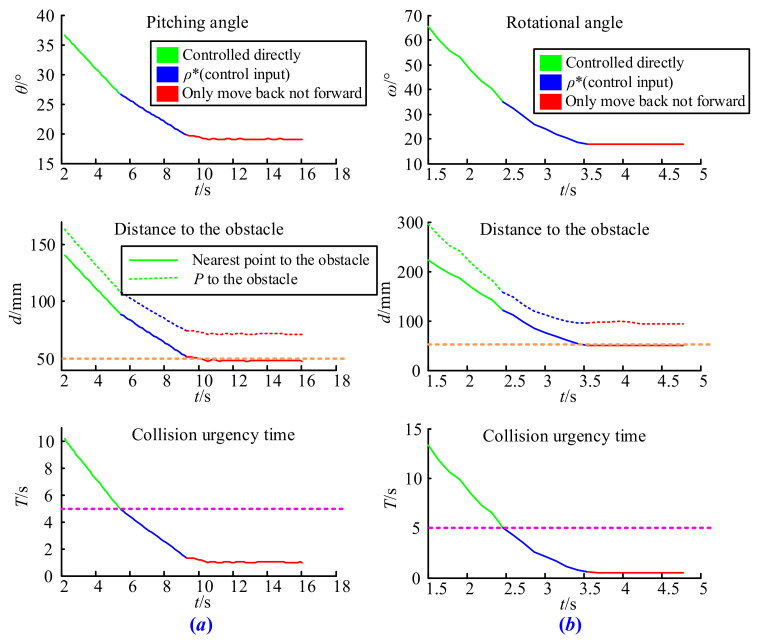
Result of the special case test: (**a**) special pitching cases, (**b**) special rotating cases.

In order to complete this test, we set *V*(180, 0, 180), *L* = 350 mm, θ=48°, and ω=0° in the pitching test, and *V*(180, 50, 130), *L* = 350 mm, θ=0°, and ω=110° in the rotating test. The results shown in Figure 13a,b once again verify the effectiveness of the collision avoidance strategy proposed in this paper. It is easy to observe that the strategy begins to work when the shortest collision time between the boom and the obstacle equals to threshold *T*_0_, and the distance from *P* to the obstacle never reaches the safety sphere in the whole process. This is because *P* is not the closest point from the boom to the obstacle. If the strategy only considers the distance between the end of the boom and the obstacle, it may still lead to a collision accident. However, after introducing special circumstances, the collision avoidance strategy guarantees that the arbitrary movement of the boom is safe.

## 5. Conclusions

In order to avoid safety accidents caused by the collision of the autocrane boom with obstacles, a collision avoidance strategy was proposed in this paper. In this strategy, both the prediction collision time and the distance to the obstacle were calculating during the movements of the boom. Based on the time and distance threshold, the movement of the boom was divided into three modes according to the urgency of the collision, which include direct control, proportional control, and the restricted approach, respectively. Additionally, the two kinds of special cases were considered to ensure that no part of the boom will break through the set security boundaries. The results of the semi-physical experiments demonstrate the correction of the collision avoidance strategy.

## Data Availability

Not applicable.
